# One-Year Clinical Outcome of Inspiron Stent in All-Comers Population (Analysis from 790 Consecutive Patients)

**DOI:** 10.1155/2020/6340716

**Published:** 2020-12-17

**Authors:** Felipe Falcão, Fabiano Cantarelli, Rodrigo Cantarelli, Flávio Mota, Manuela Navarro, Henrique Mota, Martinelly Santos, Daniel Cruz, André Sansônio, Marcelo Parente, Flávio Oliveira

**Affiliations:** ^1^Hospital Dom Helder Câmara, Cabo de Santo Agostinho, Brazil; ^2^Unidade de Cardiologia Invasiva (UCI)-Hospital Memorial São José, Rede D'Or São Luiz, Recife, Brazil; ^3^Department of Internal Medicine, University of Pernambuco, Garanhuns, Brazil

## Abstract

**Aims:**

The goal of this study was to evaluate the performance of the Inspiron^TM^ coronary stent (Scitech Medical™, Goiás, Brazil). The Inspiron^TM^ sirolimus-eluting stent uses an ultrathin L-605 cobalt-chromium alloy with a 75 *μ*m strut thickness platform coated with an abluminal biodegradable polymer. The polymer is eliminated from the body through the tricarboxylic acid cycle in 6–9 months, releasing 80% of the drug within 30 days after its deployment.

**Methods:**

It was a prospective, single-center registry. To represent clinical practice, all patients undergoing percutaneous coronary intervention were included in this registry. There were no exclusion criteria. Clinical follow-ups were performed at twelve months. The endpoints were the occurrence of all-cause death, definite stent thrombosis, and new revascularization.

**Results:**

Between November 2017 and May 2019, 790 patients were included (1067 lesions). The mean age was 60.42 ± 14.94 years, and 74.7% presented with acute coronary syndrome. Diabetes mellitus was present in 43.9% of patients, and previous myocardial infarction and previous percutaneous coronary intervention were present in 17.9% and 11.3%, respectively. Angiographic success was achieved in 99.1%. The incidence of all-cause death was 11.5% (6.2% in-hospital and 5.3% in the follow-up) and definitive stent thrombosis was 0.2%. New revascularization was performed in only 5.8% (target lesion revascularization: 2.2%; progression of disease in another lesion: 3.6%). Based on the multivariate regression analysis, only chronic renal failure was an independent predictor of adverse events (OR: 3.3; 95% CI: 1.22–8.92).

**Conclusion:**

The result of this single-center registry demonstrates the safety and excellent performance of the Inspiron^TM^ stent in daily clinical practice with a low rate of adverse cardiac events.

## 1. Introduction

A major reduction in the risk of cardiac events has been achieved with new drug-eluting stents (DES). These stents represented a development of early-generation technology, including polymers with enhanced biocompatibility (permanent or biodegradable), exclusively sirolimus-analogue active drugs, and stents with thin struts (50–100 *µ*m) composed of stainless steel, cobalt chromium, or platinum chromium. New-generation DES have higher efficacy and safety in comparison with both early-generation DES and bare metal stents [1].

The Inspiron^TM^ (Scitech Medical™, Goiás, Brazil) sirolimus-eluting stent uses an ultrathin L-605 cobalt-chromium alloy with a 75 *μ*m strut thickness platform coated with an abluminal biodegradable polymer. The polymer is eliminated from the body through the tricarboxylic acid cycle in 6–9 months, releasing 80% of the drug within 30 days after its deployment [2].

This study aims to evaluate Inspiron^TM^ in real-world scenarios in challenging conditions.

## 2. Methods

This study was an observational, prospective study at a single center. We included patients undergoing percutaneous coronary intervention (PCI) between November 2017 and May 2019.

The only inclusion criterion was patients with clear indication of PCI (symptoms of angina pectoris and/or documented ischemia or acute coronary syndrome). There were no exclusion criteria. The choice of access route, size of stents, and interventional strategy was entirely left to the judgment of the operator. Inspiron^TM^ was the only stent available in our center during the study. All procedures were performed according to standard techniques [3, 4]. The local Institutional Committee on Human Research approved the study protocol, and all patients provided written informed consent to participate.

Demographic and clinical data were recorded in a dedicated database for all patients. Procedural success was defined as achieving a minimum stenosis diameter reduction to less than 10% in the infarct-related artery, along with TIMI (thrombolysis in myocardial infarction) grade flow ≥3 without angiographic complication. TIMI flow was assessed as previously reported [5]. Patients were followed up for one year.

Aspirin was initiated before the procedure and maintained lifelong. A loading dose of clopidogrel was administered for patients not receiving these medications before the procedure. Dual antiplatelet therapy was continued for one year.

The primary endpoint was the occurrence of all-cause death. Secondary endpoints were definitive stent thrombosis (ST) and new revascularization. Definitive ST was defined as angiographic confirmation of thrombus that originates in the stent or in the segment 5 mm proximal or distal to the stent, with or without vessel occlusion, which is associated with acute onset of ischemic symptoms at rest or electrocardiographic signs of acute ischemia or typical rise and fall of cardiac biomarkers within 48 hours of angiography [6].

Continuous variables were expressed as means and standard deviations or as the median and the 25th and 75th percentiles, according to the presence or absence of a normal distribution, as evaluated by the Kolmogorov–Smirnov test. The Mann–Whitney test and Student's *t*-test were used for continuous variables in accordance by the distribution. Categorical variables were expressed as absolute numbers and percentages. SPSS software version 20 (IBM, Armonk, NY, USA) was used for all analyses, and a final *p* value less than 0.05 was considered significant.

Univariate and multivariate regression analyses were used to identify potential independent predictors of adverse events. The model was performed using forward stepwise selection. Odds ratios (OR) and their respective confidence intervals (95% CI) were used to quantify the effects.

## 3. Results

Seven hundred ninety patients were treated in our center during the study with 1376 Inspiron^TM^. The number of lesions treated was 1067.

Overall, the mean age was 60.42 (±14.94) years, 57% were male, and 74.7% presented with acute coronary syndrome (ACS). Diabetes mellitus was present in 43.9% of patients, and previous myocardial infarction and previous PCI were in 17.9% and 11.3%, respectively (Table 1).

Procedural characteristics are presented in Table 2. Left anterior descending PCI was performed in 47.1%. Directing stenting was possible in 33.9% of the lesions, and the number of stents used per lesion was 1.29 ± 0.57. Angiographic success was achieved in 99.1%.

Up to 12 months, clinical follow-up was available in 741 patients (93.7%). The incidence of all-cause death was 11.5% (6.2% in-hospital and 5.3% in the follow-up) and definitive ST was 0.2%. New revascularization was performed in only 5.8% (target lesion revascularization: 2.2%; progression of disease in another lesion: 3.6%) (Table 3). Based on the multivariate regression analysis, only chronic renal failure was an independent predictor of adverse events (OR: 3.3; 95% CI: 1.22–8.92).

## 4. Discussion

In this real-world study, without exclusion criteria, this sirolimus stent showed safety and excellent performance in daily clinical practice with a low rate of adverse cardiac events. To the best of our knowledge, this is the first study that enrolled patients with STEMI (primary PCI) for Inspiron^TM^ DES.

INSPIRON-I trial was a first-in-man evaluation of the safety and efficacy of the Inspiron^TM^. It was a multicenter, randomized trial, to compare the Inspiron^TM^ with a bare metal stent with the same metallic structure (Cronus^TM^). At six months, the in-segment late lumen loss was reduced in the Inspiron^TM^ group (0.19 ± 0.16 mm vs. 0.58 ± 0.4 mm, respectively; *p* < 0.001), as well as the percent neointimal obstruction (7.8 ± 7.1% vs. 26.5 ± 11.4%; *p* < 0.001). At four years of follow-up, there was no early or late stent thrombosis, despite the fact that INSPIRON-I trial was not powered for clinical events (only 60 lesions enrolled) [7, 8].

In the DESTINY trial, 170 patients were randomized in the ratio 2 : 1 for Inspiron^TM^ or Biomatrix^TM^ (biolimus-eluting stent), respectively. The primary endpoint of in-stent late lumen loss was not significantly different between the stents. The relatively limited sample size did not permit a formal comparison between the study groups for the risk of clinical adverse events [9].

An intravascular imaging substudy from the DESTINY trial evaluated 70 patients with intravascular ultrasound (IVUS) and 25 patients with optical coherence tomography (OCT) after 9 months. The main endpoints were percent of neointimal tissue obstruction by IVUS and neointimal strut coverage by OCT. Patients treated with both DES had very little neointimal hyperplasia either by IVUS (percent of neointimal hyperplasia obstruction of 4.9 ± 4.1% with Inspiron vs. 2.7 ± 2.9% with Biomatrix; *p*=0.03) or by OCT (neointimal thickness of 144.2 ± 72.5 *μ*m Inspiron^TM^ vs. 115.0 ± 53.9 *μ*m with Biomatrix^TM^; *p*=0.45). Regarding OCT strut-level assessment, again both devices showed excellent performance, with high rates of strut coverage (99.49 ± 1.01% with Inspiron^TM^ vs. 97.62 ± 2.21% with Biomatrix^TM^; *p* < 0.001) and very rare malapposition (0.29 ± 1.06% with Inspiron^TM^ vs. 0.53 ± 0.82% with Biomatrix^TM^; *p*=0.44). Patients with any uncovered struts were more frequently identified in the Biomatrix^TM^ group (9.78 ± 7.13 vs. 2.29 ± 3.91%; *p* < 0.001). This imaging analysis showed that both DES were effective in terms of suppressing excessive neointimal response, with very high rates of apposed and covered struts, suggesting a consistent and benign healing pattern [10].

Prado Jr. et al demonstrated low rates of events in 470 patients treated with Inspiron^TM^. At 300 days of follow-up, ST and all-cause death were 0.4 and 2.8%, respectively. Stable coronary disease was the reason for PCI in almost 60% of patients. Also, few patients were treated after a recent acute myocardial infarction, and none received stents in the context of primary PCI [2].

In comparison with other DES, Inspiron^TM^ presented rates of definitive ST and TLR similar to historical cohorts previously published (Table 4). It is important to highlight that our population was basically constituted by ACS patients when compared to other registries. The risk of adverse events is distinct to patients with stable disease, mainly in relation to mortality [11–15].

In our trial, Inspiron^TM^ was used as a workhorse stent in a center specialized in ACS. Although our all-cause death was high, it is comparable to other studies in similar situations [16–18]. A reason that could be responsible for this rate is the real-world scenario associated with ACS. The so-called off-label indications are the majority in our center. Heavy calcification, older patients, multivessel disease, and low ejection fraction are rules and not exceptions.

Some limitations must be considered. First, it was a registry in a center for ACS treatment, so extrapolation of this data should be done to patients with similar characteristics. Multicenter randomized controlled trials should be done to confirm our findings. Second, the analysis of outcomes was limited to twelve months. So, this study is not able to make any conclusions regarding very long-term prognosis.

## 5. Conclusion

In this single-center, real-world experience, Inspiron^TM^ stent proved to be safe and demonstrated excellent performance.

## Figures and Tables

**Table 1:** Baseline clinical characteristics of patients with the Inspiron^TM^.

Variables	Inspiron^TM^ (*n* = 790)
Male	451 (57.1%)
Age	60.42 ± 14.94
Hypertension	700 (89.9%)
Diabetes mellitus	347 (43.9%)
Current smoker	178 (34.2)
Family history of CAD	45 (7.4%)
Chronic renal failure	39 (5.1%)
Previous MI	137 (17.9%)
Heart failure	19 (2.5%)
Previous PCI	84 (11.3%)
Previous CABG	23 (3%)

Clinical presentation	
** **Stable angina	200 (25.3%)
** **Non-STEMI	271 (34.3%)
** **STEMI	319 (40.4 %)

Values are expressed as *n* (%) or mean ± SD. CAD: coronary artery disease; MI: myocardial infarction; PCI: percutaneous coronary intervention; CABG: coronary artery bypass graft; STEMI: ST elevation myocardial infarction.

**Table 2:** Angiographic characteristics.

	Lesions (*n* = 1067)
Target vessel	
RCA	277 (26%)
LAD	503 (47.1%)
LCX	252 (23.6%)
LM	24 (2.2%)
Bypass graft	11 (1.1%)

Number of lesions treated/patient	1.35

TIMI flow before procedure	
<2	366 (34.3%)
≥2	701 (65.7%)

Restenotic lesions	49 (4.6%)
Bifurcation involved	133 (12.5%)
Predilatation	705 (66.1%)
Numbers of stents used per lesion	1.29 ± 0.57
Total stent length per lesion	29.4 ± 16.1
Angiographic success	1058 (99.1%)

Values are expressed as *n* (%). LAD: left anterior descending coronary artery; RCA: right coronary artery; LCX: left circumflex artery; LM: left main; TIMI: thrombolysis in myocardial infarction flow.

**Table 3:** Major adverse cardiac events in twelve months.

Variables	Inspiron^TM^ (*n* = 741)
All-cause death	85 (11.4%)
Definite ST	15 (0.2%)

New revascularization	43 (5.8%)
Target lesion revascularization	16 (2.1%)
Progression of disease in another lesion	26 (3.5%)

ST: stent thrombosis.

**Table 4:** Comparison between Inspiron and other DES (1-year follow-up).

	Inspiron^TM^ current registry	Promus^TM,12^	Synergy^TM,13^	Resolute Onyx^TM,11^	Ultimaster^TM,14^
Number of patients	790	988	846	252	1660
Diabetes	43.9%	35.2%	31.1%	46.2%	29%
Non-STEMI	34.3%	7.3%	25.9%	24.4%	12.5%
STEMI	40.4%	5.8%	0	8.8%	10.5%
Stent thrombosis	0.2%	0.5%	0.4%	0.7%	0.9%
Target lesion revascularization	2.1 %	1.9%	2.0%	4.4%	3.2%

## Data Availability

The datasets generated during the current study are available in the https://figshare.com/ repository, named as Corehemo-data.
